# Enhancement of tumor lethality of ROS in photodynamic therapy

**DOI:** 10.1002/cam4.3592

**Published:** 2020-11-03

**Authors:** Lan Ming, Kai Cheng, Yu Chen, Rui Yang, Daozhen Chen

**Affiliations:** ^1^ Research Institute for Reproductive Health and Genetic Diseases The Affiliated Wuxi Maternity and Child Health Care Hospital of Nanjing Medical University Wuxi China

**Keywords:** hypoxia, photodynamic therapy, reactive oxygen species, ROS defense system, tumor treatment

## Abstract

In the process of photodynamic therapy (PDT) treatment of tumors, reactive oxygen species (ROS) plays a key role in destroying tumor tissues. However, traditional PDT often has limited ROS killing capacity due to hypoxia in the tumor microenvironment (TME) or obstruction by the ROS defense system, resulting in poor efficacy. Therefore, enhancing the killing effect of ROS on tumors is the core of enhancing the anti‐tumor effect of PDT. In recent years, many studies have developed a series of strategies to enhance the ability of ROS to kill tumors in view of the limitations of the TME on PDT. This article summarizes the commonly used or innovative strategies in recent years, including not only frequently used methods for hypoxia in the TME but also innovative strategies to inhibit the ROS defense system.

## Graphical abstract

In recent years, many studies have developed a series of strategies to enhance the ability of reactive oxygen species (ROS) to kill tumors in view of the limitations of the tumor microenvironment (TME) on photodynamic therapy. This article summarizes the commonly used or innovative strategies in recent years, including not only the common methods for TME hypoxia but also innovatively covering the relevant strategies for inhibiting the ROS defense system.

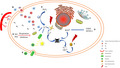

## 1 INTRODUCTION

Photodynamic therapy (PDT) is an emerging tumor treatment, which has received more and more attention in the field of tumor treatment due to its advantages of low invasiveness, low systemic toxicity, no drug resistance, and high treatment efficiency.^1^, ^2^, ^3^, ^4^, ^5^ PDT is divided into two types, Ⅰ and Ⅱ. The type Ⅰ pathway mainly generates superoxide radicals (O_2_^●−^), hydroxyl radicals (^●^OH), and other cytotoxic reactive oxygen species (ROS) through hydrogen or electron transfer. Therefore, the biggest advantage of type Ⅰ PDT is that it can also occur in an oxygen‐deficient environment without being restricted by oxygen concentration.^6^, ^7^, ^8^ However, most of the PDTs related to clinical applications are based on the type Ⅱ pathway, requiring the participation of oxygen, photosensitizers (PS), and light sources.^9^ Under the irradiation of a specific wavelength light source, the PSs are activated and transfer their energy to molecular oxygen,^10^, ^11^ which convert oxygen from the ground state to a cytotoxic singlet state, thereby generating singlet oxygen (^1^O_2_) that has a killing effect on tumor cells.^12^, ^13^, ^14^, ^15^ Therefore, ROS plays a key role in killing tumors in the process of PDT. ROS mainly includes superoxide anion (O_2_^●−^), hydroxyl radical (^●^OH), and singlet oxygen (^1^O_2_).^16^ The physiological level of ROS plays a role in signal transduction in cells, but when the level of ROS is abnormally elevated, it can cause irreversible cell damage.^17^ ROS generated during PDT oxidizes biological macromolecules such as DNA/RNA and protein in tumor cells, thereby inducing tumor cell apoptosis. In addition, ROS can also cause microvascular damage and immunogenic cell death (ICD) in tumor tissue.^18^, ^19^, ^20^ It can be seen that the therapeutic effect of PDT depends on the tumor lethality of ROS.

**SCHEME 1:**
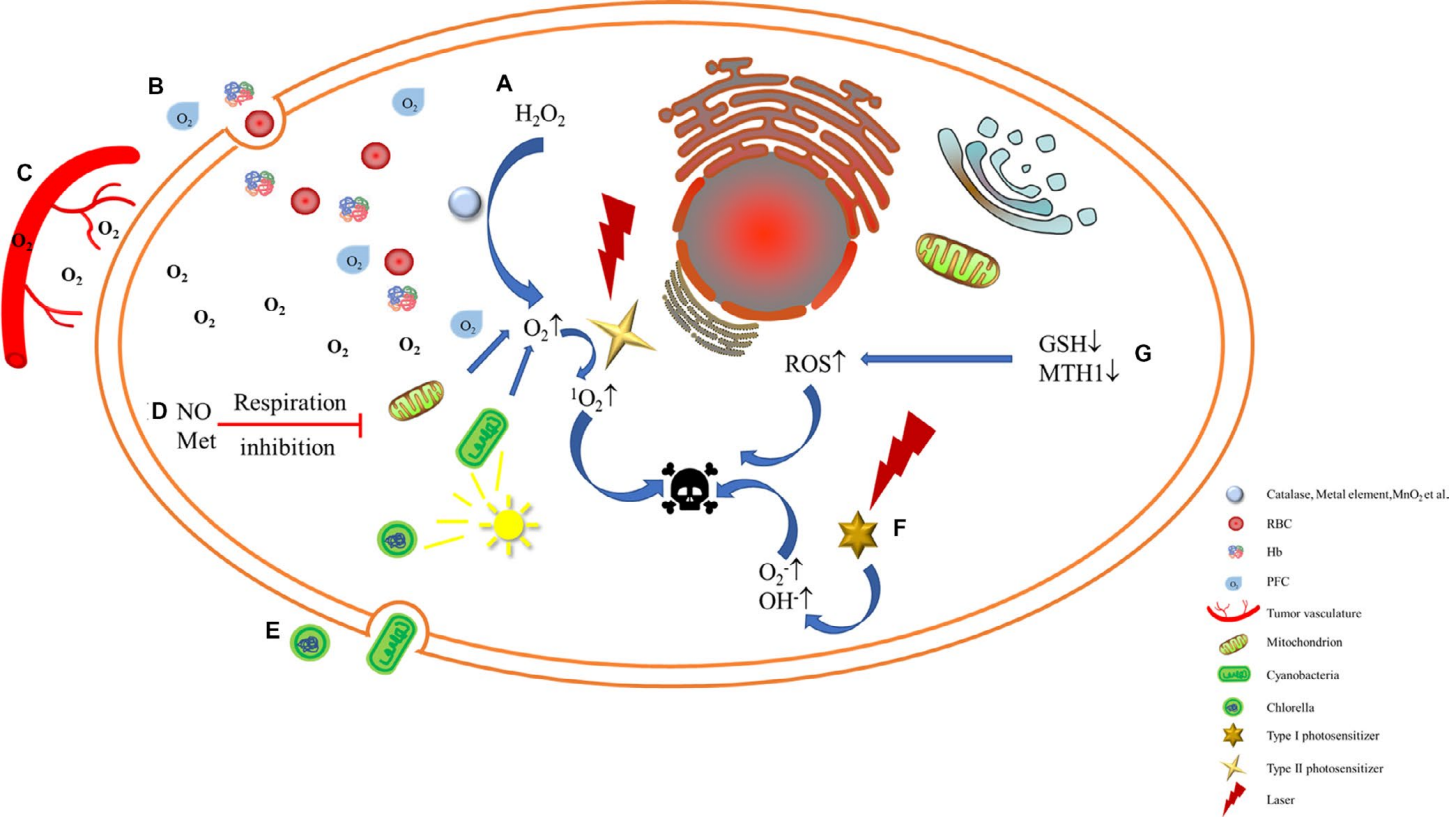
Schematic illustration of enhancing tumor lethality of reactive oxygen species (ROS) in photodynamic therapy (PDT). (A) Decompose hydrogen peroxide to increase oxygen content. (B) Oxygen delivery. (C) Normalization of tumor vasculature. (D) Reduce the tumor's own oxygen consumption. (E) Photosynthesis of microorganisms. (F) Type Ⅰ PDT. (G) Inhibition of ROS defense system.

Although PSs have been approved by the FDA for clinical treatment of tumors, PDT has not yet become the first‐line treatment for tumors. PDT has certain limitations that restrict its development.^21^, ^22^, ^23^, ^24^ Most PDTs rely on a large amount of oxygen in the process of achieving the destruction of tumors. Unfortunately, the tumor tissue is in a low oxygen state and cannot provide enough oxygen for PDT.^25^ Furthermore, the ROS defense system in the tumor microenvironment (TME) weakens the anti‐tumor ability of ROS.^26^, ^27^ Therefore, the TME largely limits the killing effect of ROS on tumors, making it difficult for the efficacy of PDT to achieve the expected results. In the past few decades, in response to the limitations of the TME, researchers have developed a series of strategies to enhance the killing effect of ROS on tumor tissues, including strategies to increase the level of ROS production and inhibit the ROS defense system. Among them, the strategy of improving the lethality of ROS by alleviating hypoxia in the TME has been elaborated.^18^, ^28^, ^29^ In this review, as shown in Scheme 1, in addition to describing the latest progress in alleviating hypoxia to enhance ROS production, it also involves strategies to inhibit the ROS defense system, summarizing both from promoting ROS production and inhibiting its consumption. Besides, this article makes prospects for the challenges and future development of PDT, providing a reference for follow‐up research to promote the future clinical translation and application of PDT.

## 2 IMPROVE ROS GENERATION LEVEL

The killing effect of ROS in tumor tissues is closely related to the production of ROS.^9^ Once the output of ROS is increased, the killing effect of ROS on tumors can be greatly improved. Oxygen is used as the raw material for ROS generation, which determines the level of ROS generation. However, due to excessive proliferation and insufficient blood supply, the TME is in a hypoxic condition (pO_2_ values 2.5 mmHg),^30^, ^31^, ^32^, ^33^ which limits the generation of ROS and affects the ability of ROS to kill tumors.^9^, ^34^, ^35^ At the same time, PDT also consumes a lot of oxygen while generating ROS, which aggravates the hypoxia of tumor tissues, further reducing the efficacy of PDT.^2^, ^36^ Therefore, the restriction of ROS generation can be solved by relieving the hypoxia of tumor tissue, which is of great significance for enhancing the killing of ROS on tumors. Researchers have developed a series of strategies to alleviate hypoxia in tumor tissues, which effectively improve the generation and tumor lethality of ROS, laying a foundation for improving the efficacy of PDT. Moreover, researchers also found a method of generating ROS independent of oxygen, which provides a new way of thinking to get rid of the hypoxia of tumor tissue.

### 2.1 Relieve tumor tissue hypoxia

#### 2.1.1 Decompose hydrogen peroxide to increase the oxygen content

Studies have shown that tumor tissue contains higher levels of H_2_O_2_ than normal tissue.^37^, ^38^, ^39^ Catalase can catalyze the decomposition of H_2_O_2_ into water and oxygen. Based on this feature, researchers devised a method to generate a large amount of oxygen by decomposing H_2_O_2_ in situ of tumor tissue to alleviate the hypoxic condition inside the tumor. Shen et al.^40^ combined catalase and chitosan through electrostatic interactions and encapsulated the PS Chlorin 6 (Ce6) in nanoparticles, successfully preparing pH‐responsive oxygen self‐sufficient nanoparticles. The experimental results show that the nanoparticles decompose in the acidic environment of the tumor, quickly release the catalase and PS therein, effectively alleviate the hypoxia of the tumor tissue, and continuously form cytotoxic singlet oxygen under the laser, thereby enhancing the killing effect of ROS on tumors. The flow cytometry result shows that the apoptosis rate induced by C&C‐Ce6 nanoparticles is 94.31%, which is higher than that of free Ce6 (78.57%). *In vivo* level, 650 nm laser was used to irradiate the tumor area of CAL‐27 tumor‐bearing mice for 10 min (12 J/cm^2^). The body weight and tumor volume of the mice were continuously monitored. The results confirm that compared with other preparations, C&C‐Ce6 nanoparticles have obvious tumor growth inhibitory effects. Both *in vitro* and *in vivo* biological studies have confirmed that the nanoparticles can greatly enhance the efficacy of PDT in hypoxic tumor tissues.

In addition, researchers found that MnO_2_ nanoparticles,^41^ gold nanoclusters,^42^ platinum nanozymes,^43^ etc. can be used to decompose H_2_O_2_. For example, Cao et al.^44^ loaded MnO_2_@Ce6 nanoparticles into mesenchymal stem cells (MSC) and transported them to the tumor site. The massive absorption of MSCs by tumor tissues increases the accumulation of the nanoplatforms (MnO_2_@Ce6‐MSCs) inside the tumor. MnO_2_ generates O_2_ in situ by reacting with tumor endogenous H_2_O_2_ under acidic conditions, thereby regulating the hypoxic TME. The 2′,7′‐Dichlorofluorescin diacetate (DCFH‐DA) fluorescent probe was used to directly measure the total generation rate of ROS in flow cytometry. The result shows that the fluorescence intensity of MnO_2_@Ce6 nanoparticles is significantly enhanced, which confirms that the increased oxygen of MnO_2_@Ce6 could be converted into ROS. Therefore, the O_2_ produced by the decomposition of H_2_O_2_ under 633 nm laser irradiation can serve as a rich source of ^1^O_2_ generation, overcoming the key bottleneck of TME's hypoxic conditions that are not conducive to PDT. Furthermore, MnO_2_ is decomposed into Mn^2+^, which is manifested as high T1 relaxation in magnetic resonance imaging (MRI). Therefore, the nanoplatform can enhance the killing efficiency of ROS on tumors by alleviating the hypoxia of tumor tissues, and can also realize MRI monitoring.

The above substances used to decompose H_2_O_2_ are more common in PDT research. Besides, the researchers have discovered some new strategies. The nanoplatform constructed by Yang et al.^45^ was composed of Prussian blue (PB) core and Ce6 anchored periodic mesoporous organosilica (PMO) shell. PB with catalase‐like activity can catalyze hydrogen peroxide to generate O_2_, and Ce6 can convert newly generated O_2_ into ^1^O_2_ during laser irradiation to generate more ROS. After 660 nm laser irradiation, the ^1^O_2_ level of PB@PMO‐Ce6 with or without diluted H_2_O_2_ is measured. Obviously, in the presence of Pb@PMO‐Ce6 and H_2_O_2_, the generation rate and total amount of ^1^O_2_ are higher than PB@PMO‐Ce6 only, which proved the ability of PB@PMo‐Ce6 to generate more active oxygen. The use of Singlet Oxygen Sensor Green (SOSG) probe *in vivo* found that PB@PMO‐Ce6‐treated tumors showed stronger green fluorescence than Ce6‐treated tumors, which also indicated that PB@PMO‐Ce6 achieved higher singlet oxygen production *in vivo*. The results of this study show that the singlet oxygen level and therapeutic effect of the PB@PMO‐Ce6 group are higher than those of the Ce6 group, both *in vivo* and *in vitro*, indicating that the nanosystem has the ability to enhance PDT. Moreover, Wang et al.^46^ employed MOF as a single‐atom anchoring material and successfully prepared a multifunctional OxgeMCC‐r single‐atom enzyme (SAE) through a one‐step self‐assembly strategy. In OxgeMCC‐r SAE, single‐atom ruthenium is anchored on the metal‐organic framework Mn_3_[Co(CN)_6_]_2_ as the active catalytic center, and Ce6 is encapsulated. Ru partly replaces Co as a single‐atom catalytic center to generate oxygen. When endogenous H_2_O_2_ reacts with single‐atom Ru species of OxgeMCC‐r SAE to generate O_2_, the hypoxia in the TME can be alleviated. Hypoxia‐inducible factor (HIF)‐1α staining shows that after Mn_3_[Co(CN)_6_]_2_‐Ru treatment, as the concentration increased from 25 to 50 ppm, the green fluorescence decreases, and the expression of HIF‐1α is significantly down‐regulated. The results of the MTT experiment show that, under hypoxic conditions, the PDT cytotoxicity of the free Ce6 group and the MC‐Ce6 group is only 18.4% and 25.3%, respectively. When incubated with OxgeMCC‐r (26.7 ppm) and then irradiated with 671 nm laser, nearly 90% of cancer cells are killed. Additionally, the subject also finds that under the same active component concentration (0.5 ppm Ru for OxgeMCC‐r and 0.5 ppm Mn for MnO_2_), the catalytic reaction rate constant of OxgeMCC‐r is higher than that of the widely used MnO_2_. Hence, one can see that OxgeMCC‐r SAE, due to its higher hydrogen peroxide catalytic efficiency, is expected to become an effective way to solve tumor hypoxia.

In summary, the decomposition of H_2_O_2_ in tumor tissue can significantly alleviate hypoxia in tumor tissue and increase the generation of ROS, which has become an important strategy to enhance the killing effect of tumors by ROS. More and more nano‐enzymes that can be used to decompose H_2_O_2_ have been discovered, and some can also have functions such as imaging, laying the foundation for the construction of multifunctional PDT nanomaterials.

#### 2.1.2 Oxygen delivery

As the oxygen transport cells in the human body, red blood cells have good biocompatibility. Inspired by this, the researchers used red blood cells to deliver oxygen into tumor tissues to increase the local oxygen content of the tumor. Tang et al.^47^ introduced RBC into PDT and used RBC to deliver oxygen to tumor tissues to relieve tumor hypoxia. RBC can also be served as a carrier to deliver PS to tumor tissue. Since the PS is adjacent to the O_2_ source carried by RBC, it can also effectively produce ^1^O_2_ under hypoxic conditions, thereby enhancing the killing ability of ROS. This kind of PDT combined with RBC to relieve tumor hypoxia showed good tumor suppression. At present, the commonly used bionic red blood cells are not able to deliver large amounts of oxygen to tumor tissues, but also have good biocompatibility, showing a long blood circulation time in the organism.^48^, ^49^ Liu et al.^50^ have engineered a type of biomimetic pseudo‐RBCs (AmmRBC) for oxygen self‐supplied PDT to tumors by encapsulating hemoglobin (Hb), PDA, and PDA‐adsorbed PS inside biological vesicles transformed from recombinant RBC membranes. Due to the same origin of the outer membrane, AmmRBCs inherit the good biocompatibility of RBCs. Moreover, the average Hb content of AmmRBC is about 10 times that of natural RBC, so it has a strong oxygen storage capacity. This kind of bionic red blood cells can accumulate in tumor tissues and supply large amounts of oxygen to the hypoxic tumor tissues. The DCFH‐DA probe can produce green fluorescence when interacting with ^1^O_2_. Confocal laser microscope was used to detect the generation of ^1^O_2_ in the cells, and the order of green fluorescence intensity was MB<MB plus RBC≈MB plus mmRBC<AmmRBC. HIF‐1α staining gave similar results. It can be seen that AmmRBC exhibits a strong radiation activation ability to generate ^1^O_2_, which improves the level of ROS generation during PDT. The anti‐tumor effect of AmmRBC *in vivo* was evaluated on the 4T1 mouse model. The results show that the tumor is completely eliminated. Compared with the control group, the AmmRBC group had an excellent effect on PDT. In summary, AmmRBC can increase the supply of oxygen during PDT, thereby significantly improving the efficacy of PDT. It can also be used as a general self‐supplying platform to sensitize other hypoxia‐limited treatments, such as chemotherapy and radiotherapy.

Hemoglobin is responsible for transporting oxygen in RBC. Hb can reversibly bind four oxygen molecules and is widely used as a biologically safe oxygen carrier in oxygen‐enhanced PDT.^51^, ^52^ Chen et al.^53^ assembled human serum albumin and Hb together to develop a hybrid protein oxygen nanocarrier that encapsulates Ce6 (C@HPOC) for oxygen self‐sufficiency PDT. In order to verify whether C@HPOC can effectively deliver oxygen to tumor oxygenation, the study used photoacoustic (PA) imaging to measure HbO_2_ signals to monitor the corresponding results. The experimental results suggest that, compared with the control group, the PA intensity of HbO_2_ around the blood vessel in C@HPOC increases significantly at 2–6 h, and the peak value of the PA intensity of HbO_2_ appears at 4 h, which is about 47% higher than before the injection. *In vivo* experiments prove that C@HPOC helps to improve the efficiency of PDT to a large extent. Therefore, C@HPOC realizes the delivery of PS and oxygen on the tumor and significantly relieves the tumor's hypoxia. It is also worth noting that in the metastatic triple‐negative breast cancer model, C@HPOC can destroy the primary tumor and effectively inhibit distant metastasis by evoking the system's anti‐tumor immunity. This study provides an example of aerobic immunogenic PDT for the treatment of metastatic cancer.

Besides, there is a commonly used oxygen carrier widely used in PDT research, namely, perfluorocarbon (PFC). The high electronegativity of fluorine makes PFCs own excellent oxygen affinity.^54^ FDA has approved PFC materials (such as C5F12) to directly transport oxygen molecules.^55^ Shen et al.^56^ combined perfluorooctyl bromide (PFOB) with ICG nanoliposomes as a carrier through a two‐step emulsion method to prepare ICG&PFOB co‐loaded nanoliposomes (LIP‐PFOB‐ICG). PFOB has excellent oxygen‐carrying capacity, which effectively reduces the hypoxia of the tumor and improves the killing effect of ROS on the tumor. LIP‐PFOB‐ICG completely inhibits the growth of MDA‐MB‐231 tumors through enhanced PDT and photothermal therapy (PTT) synergistic treatment, and comprehensively enhances the efficiency of ICG for PDT treatment. Furthermore, studies have shown that the lifespan of ROS generated in PFC is significantly longer than that in the cellular microenvironment.^28^

The above are common oxygen supply carriers, which play an important role in the research of alleviating hypoxic PDT. However, with the development of technology, more and more new materials are found to be used for oxygen supply. Song et al.^57^ explored the potential of biogenic nanobubbles vesicles (GVs) as a new type of oxygen carrier to relieve tumor hypoxia. Among them, GV is a naturally formed oxygen carrier, and lipid‐GVS(O_2_) is prepared by modifying the liposome layer on the surface of its protein shell. Observe the levels of oxy‐Hb and deoxy‐Hb by PA imaging to monitor the oxygenation of the tumor. The result shows that lipid‐GVS(O_2_) can increase the oxygenation level of hypoxic tumors in the body after 5 min of tail injection, reaching a peak after 15 min of injection, and then gradually decrease, indicating that it has a significant oxygen transport capacity. *In vivo* and *in vitro* PDT measurements suggest that the lipid‐GVS(O_2_) group shows effective tumor growth inhibitory effects. In short, the high oxygen replenishment capacity and low toxicity of this biogenic nanobubbles bubble make it have bright prospects in clinical application. Besides, there are reports in the literature that the metal‐organic framework is also used to deliver oxygen to tumor tissues due to its significant gas adsorption capacity.^58^

#### 2.1.3 Normalization of tumor blood vessels

Abnormal vasculature and blood vessel closure in tumor tissue are important factors for hypoxia in the tumor, which seriously hinders the efficacy of PDT. Therefore, normalizing tumor blood vessels is an effective method to improve the oxygenation of tumor tissue and enhance PDT. Studies have found that heating the tumor tissue in a warm water bath at 43°C can increase the blood flow of the tumor and increase the oxygen level inside the tumor. Hu et al.^59^ developed a thermally regulated ROS strategy that promotes anticancer ability of PDT through human serum albumin‐Ce6 nanocomponents (HSA‐Ce6 NAs). By accurately controlling the overall body temperature, instead of increasing the local tumor temperature from 37–43°C, the oxygen saturation of the tumor tissue increased by 52%, which significantly enhanced the ROS generation that promotes PDT. Inspired by heating, PTT has also been applied to improve tumor tissue vasculature. Local heat generated by PTT promotes the delivery of PSs within the cell, thereby increasing the production of ROS.^60^ Additionally, PTT also increases the blood flow in the tumor, enriches the oxygenation of the tumor, and effectively improves the efficacy of PDT.^61^ Feng et al.^62^ co‐wrapped hCe6 and dir molecules in bilayers of polyethylene glycol (PEG) shelled liposomes and constructed a near‐infrared (NIR) light‐activated liposome (DiR‐hCe6‐liposome). The as‐prepared DiR‐hCe6‐liposome was injected intravenously into mice and irradiated with 785 nm NIR laser, which caused mild photothermal heating, promoting the increase in blood flow in the tumor and relieving the hypoxia of the tumor. Using pimonidazole as a hypoxia staining probe to perform immunofluorescence staining on tumor tissues, it is found that 4T1 tumors showed obvious hypoxia, but the hypoxia can be significantly relieved after NIR photothermal heating treatment of DiR‐hCe6‐liposom. Semi‐quantitative analysis of the hypoxia‐positive signal in these tumor slices finds that the percentage of hypoxia‐positive area drops sharply from 38% to only 12%, confirming the improvement of hypoxia by the photothermal effect.

Gong et al.^63^ discovered a strategy to promote the normalization of tumor blood vessels by hyaluronidase (HAase) to relieve hypoxia in tumor tissues. HAase can decompose hyaluronic acid (HA) in the extracellular matrix of tumors, increase the permeability of drugs,^64^ and at the same time reduce the flow pressure of the tumor tissue gap. Additionally, the HA degradation product 4–25 disaccharide unit oligosaccharide is one of the angiogenic molecules,^65^ which causes the percentage of tumor‐dilated blood vessels and the density of the blood vessels to increase, and promotes the normalization of the vasculature in the tumor and the increase in the perfusion of the tumor. The experimental result indicates that the uptake of nanomicelles (NM‐Ce6) covalently linked to Ce6 increases by 2 times in the tumor, and the result of HIF‐1α staining shows that the oxygenation level of the tumor is significantly increased, and the hypoxic state in the tumor is effectively eased. Therefore, HAase promotes more ROS production during PDT, which enhances the efficacy of PDT for cancer treatment *in vivo*.

Recently, Zhu^66^ and others have prepared tumor‐exocytosed exosome/AIEgen hybrid nanovesicles (termed DES) that can promote effective tumor penetration using electroporation to achieve targeted photodynamic killing of tumor tissue. Besides, dexamethasone is used to normalize the vascular function in the TME, thereby alleviating the problem of tumor hypoxia, and significantly enhancing the PDT efficacy of DES nanovesicles. It can be seen that the normalization of tumor tissue blood vessels can overcome the limitations of tumor tissue hypoxia on PDT, making a great contribution to the improvement of the efficacy of PDT.

#### 2.1.4 Reduce the tumor's own oxygen consumption

Although the above‐mentioned treatment plans have effectively alleviated the predicament of hypoxia and greatly improved the anti‐tumor ability of PDT, they still face huge challenges, including low O_2_ production efficiency, premature O_2_ leakage, and insufficient tumor microvessels. Therefore, it is necessary to develop new strategies to improve tumor oxygenation from other perspectives to improve the efficiency of PDT. Reducing the oxygen consumption of tumor tissue provides a good idea for the development of new strategies. Zhao et al.^67^ developed an O_2_‐economized PDT strategy for oxygen‐deficient tumors by preparing a self‐delivery nanodrug (called ACSN) composed of Oxidative phosphorylation (OXPHOS) inhibitor of atovaquone (ATO) and Ce6. OXPHOS metabolic pathway requires continuous consumption of O_2_ to produce ATP,^68^, ^69^, ^70^ and ATO reduces oxygen consumption of OXPHOS by inhibiting mitochondrial complexes in the mitochondrial electron transport chain, which can alleviate hypoxia in tumor tissues.^71^, ^72^, ^73^ 4T1 cells were incubated with ATO or ACSN, and then mitochondria were labeled with rhodamine 123. The mitochondrial function shows bright green fluorescence when it is not severely impaired. However, the fluorescence of cells treated with ATO or ACSN is significantly reduced, confirming that both ATO and ACSN inhibit the mitochondrial complex. Furthermore, 4T1 tumor‐bearing mice were injected intravenously with ACSN for PA imaging detection. The results show that the PA signal gradually goes up, suggesting an increase in O_2_ content in the tumor. Quantitative analysis shows that O_2_ content in tumor tissues increases 1.6 times compared with the control group. Therefore, ACSN successfully demonstrates a powerful tumor suppressor effect, providing an alternative strategy for the development of PDT in hypoxic tumors. Similarly, Yu et al.^74^ incorporated nitric oxide (NO) and PSs into poly(D,L‐lactide‐co‐glycolide) nanovesicles, and saved endogenous sources by inhibiting the O_2_ consumption of tumor cell respiration by NO, thereby overcoming the hypoxic barrier of tumor tissue. What is surprising is that metformin (Met) can also help enhance the efficacy of PDT by alleviating hypoxia in tumor tissues. Mai et al.^75^ encapsulated Met and IR780 (constructed PM‐IR780‐Met NP) with platelet membrane (PM) as a nanocarrier. The effective adhesion between PM and tumor cells can ensure the longer cycle life of PM‐IR780‐Met NPs and promote the greater accumulation of IR780 and Met in tumors. Met reduces oxygen consumption in tumors by inhibiting mitochondrial respiration, which indirectly increases oxygen content and enhances the ability of ROS to kill tumors. *Ex vivo* HIF‐1α staining, *in vivo* PA imaging, and *in vivo* positron emission tomography (PET) imaging were used to detect the effect of Met on inhibiting mitochondria and hypoxia. Among them, the PM‐IR780‐Met NP group has the lowest fluorescence intensity, indicating that the expression of HIF‐1α is the lowest and the degree of hypoxia is the mildest. The PA imaging results show that the PA signal of HbO_2_ in the PM‐IR780‐Met NP group is the strongest, and the PA signal of Hb is the lowest, suggesting that Met can effectively inhibit mitochondrial respiration and significantly improve tumor hypoxia. ^18^F fluoromisonidazole (FMISO) PET probe is highly sensitive to hypoxia, and no hypoxic area is detected at the tumor site in the PM‐IR780‐Met NPs group. It follows that Met has a surprising effect in alleviating hypoxia.

#### 2.1.5 Photosynthesis of microorganisms

In recent years, in addition to the above‐mentioned commonly used strategies to alleviate hypoxia in the TME, the method of increasing oxygen content by means of photosynthesis of microorganisms has also been used to regulate the hypoxia of tumors. Cyanobacteria photosynthesizes through chlorophyll molecules on thylakoid membranes, producing large amounts of oxygen molecules. PDT performed after the hybridization of cyanobacteria and PSs may have good prospects in enhancing the killing effect of ROS on tumors. Huo et al.^76^ constructed photodynamic cyanobacteria (ceCyan cell) synthesized by injecting clinically recognized PS Ce6 into cyanobacteria cells. Under the irradiation of 660 nm laser, cyanobacteria can continuously release O_2_ by photosynthesis, and activate the O_2_ into singlet oxygen through the injected PS. The intracellular hypoxia indicator [Ru(dpp)_3_]Cl_2_ and SOSG probe fluorescence results show that due to the photosynthesis of ceCyan cell, intratumoral hypoxia is alleviated, and the singlet oxygen generation rate is significantly increased. Moreover, combined imaging of PA and B‐mode ultrasound in oxygen saturation mode (SO_2_) was used to evaluate the oxygenation effect in the tumor. Before the injection of cyanobacteria, the observed SO_2_ value averaged 4.48%, suggesting that the transplanted TME was hypoxic. After intratumoral injection of cyanobacteria, the saturated oxygen partial pressure rose steadily to 7.71% within 5 min, and the saturated SO_2_ rose to 17.1% 20 min after injection. It further increased to 25.2% after 30 min of injection, confirming the continuous oxygen production effect of ceCyan cells in the body. This cascade process will continue to generate a large amount of ROS, which can effectively destroy tumor tissues *in vivo* and *in vitro*, and provides a new development direction for enhancing the killing effect of ROS on tumors. In addition, chlorella has also been used to relieve hypoxia in tumor tissues. Its chloroplast is rich in photosynthetic pigments chlorophyll a and chlorophyll b, which is a promising source of oxygen. Zhou et al.^77^ designed an autotrophic light‐triggered green affording oxygen engine (ALGAE), which is formed by cross‐linking chlorella and calcium alginate, and has good biocompatibility and degradability. ALGAE is implanted around the tumor tissue through a minimally invasive method because calcium alginate protects Chlorella from macrophages. Among them, the light can not only stimulate the PDT but also the energy source of Chlorella to produce oxygen. ALGAE can continue to produce a large amount of oxygen in the body after being irradiated by the light source, which can significantly relieve the hypoxia of the tumor. At the same time, oxygen continuously receives the energy transferred from the excited PS to generate sufficient singlet oxygen, thereby enhancing the ability of ROS to damage tumor tissues and the efficacy of PDT. Long‐term repeated oxygen supplementation provides a guarantee for efficient and repeated PDT treatment. The strategies for alleviating tumor hypoxia mentioned above were summarized in Table 1.

**TABLE 1:** A summary of overcoming hypoxia.

Types	Mechanism	Representative	Reference
Decompose hydrogen peroxide	Generate oxygen by decomposing hydrogen peroxide in the TME	Catalase	40
		Metal element	42, 43, 46
		MnO_2_	41, 44
		PB	45
Oxygen transportation	Transport exogenous oxygen to tumor tissue	RBC	47, 50
		Hb	53
		PFC	56
		Biogenic nanobubbles	57
Normalization of tumor blood vessels	Increase blood supply in tumor tissues and increase oxygen delivery	Warm water bath	59
		PTT	62
		HAase	63
		Dexamethasone	66
Reduce tumor oxygen consumption	Alleviate hypoxia in tumor tissue by reducing tumor's own oxygen consumption	Inhibiting mitochondrial respiration	67, 74
		Met	75
Photosynthesis	Oxygen is produced through the photosynthesis of microorganisms	Cyanobacteria	76
		Chlorella	77

Abbreviations: HAase, hyaluronidase; Hb, hemoglobin; PB, Prussian blue; PFC, perfluorocarbon; PTT, photothermal therapy; TME, tumor microenvironment.

### 2.2 Oxygen‐free generation of ROS

Facing the huge challenge of insufficient ROS killing capacity caused by hypoxia in the TME, researchers tried to develop an oxygen‐independent ROS generation strategy, breaking the limitation of the TME hypoxia condition on the killing effect of ROS from another perspective. As mentioned in the introduction, the occurrence of type Ⅰ PDT mainly depends on the transfer of electrons or hydrogen atoms, and the subsequent disproportionation reaction or Haber–Weiss/Fenton reaction can compensate for the oxygen consumption.^78^, ^79^, ^80^ Therefore, type Ⅰ PDT is less dependent on oxygen and can still produce a large amount of ROS in the hypoxic TME. In type Ⅰ PDT, superoxide radical is one of the most important ROS and the main source of other important ROS, and its effect of DNA chains break, membrane injury, and OXPHOS of mitochondria make type Ⅰ PDT have high anti‐tumor efficiency. According to the literature, Zhang et al.^81^ prepared a ZnO nanorod that delivers DNR to tumors. The ZnO nanorods which enter the tumor tissue achieve the cancer cell damage mediated by ROS through the type Ⅰ PDT pathway. So far, the traditional superoxide radical photogenerators, such as ZnO, TiO_2_, methylene blue, in type Ⅰ PDT have shown significant superoxide radical generation efficiency. Unfortunately, these PSs need to be improved in terms of biocompatibility and photostability. In recent years, boron difluoride dipyrromethene (BODIPY) has proven to be a promising type Ⅰ PSs, which can significantly improve the generation of ROS in a low oxygen environment.^82^ Chen et al.^83^ designed a highly effective type Ⅰ PS PBV NPs, which consists of BODIPY‐vadimezan conjugate (BDPVDA) and mPEG‐PPDA. The core‐shell intermolecular electron transfer between BDPVDA and mPEG‐PPDA enables PBV NP to generate a large amount of ROS under NIR radiation in a severe hypoxic environment (2% O_2_), thereby achieving effective hypoxic tumor elimination.

The application of semiconductor nanomaterials in type Ⅰ PDT has successfully promoted the development of oxygen‐independent PDT. Among them, a highly efficient oxygen‐independent PS is the key to overcome hypoxia and enhance the killing ability of ROS. Liu et al.^84^ prepared an effective inorganic PS by incorporating plasmonic gold metal nanostructures into Cu_2_O semiconductors. By utilizing the plasmon‐induced resonance energy transfer process from Au to Cu_2_O, the PS shows high singlet oxygen yield under laser irradiation. Fan et al.^85^ constructed an excellent hybrid nanocomposite composed of metal (Au deposition) and semiconductor (CdSe‐seeded/CdS nanorods). The composite material provides a reaction site for efficient water decomposition, thereby generating a large amount of ROS without the participation of oxygen. The test results of DCFH‐DA proved that the nanomaterial does not rely on oxygen to generate ROS with high efficiency. *In vitro* studies have shown that the material has high ROS generation efficiency under both aerobic and anaerobic conditions. *In vivo* studies have also shown that the material has significant tumor‐suppressing ability. Wang et al.^86^ used Cu_2_‐xSe nanoparticles modified with polyethylene glycol, β‐cyclodextrin, and Ce6 to make ultra‐small nano‐agents. The resulting nanoplatforms (CS‐CD‐Ce6 NPs) can complete the Fenton‐like Haber–Weiss catalyst, degrade H_2_O_2_ in the tumor under hypoxic conditions, release hydroxyl radical (^●^OH) and highly toxic singlet oxygen (^1^O_2_), thereby effectively improving the level of ROS generation. A large amount of ROS produced not only kills the primary tumor cells but also triggers ICD, which greatly improves the efficacy of PDT.

## 3 INHIBITION OF ROS DEFENSE SYSTEM

When some PSs are used in PDT, even if the ROS generation ability is very strong, the therapeutic effect is far from reaching the ideal state. This is due to the presence of a defense system in the tumor tissue that resists the killing effect of ROS to protect the tumor cells. Therefore, effectively inhibiting the ROS defense system will help enhance the ability of ROS to kill tumors. Studies have found that there are abundant anti‐oxidants in tumor tissues, especially the overexpression of glutathione (GSH). GSH neutralizes the ROS produced by PDT and, thus, weakens the killing effect of ROS on tumors, greatly reducing the anti‐tumor efficiency of ROS.^87^, ^88^, ^89^ The researchers realized the importance of inhibiting the ROS defense system in the PDT process, so they developed a strategy to reduce GSH in tumor tissues to enhance the ROS killing effect, and opened a new path for improving the efficacy of PDT. According to reports, copper and GSH can react through metal reduction and promote the pro‐oxidant effect.^90^ Ju et al.^91^ integrated Cu^2+^ with graphitic carbon nitride nanosheets (g‐C3N4), successfully constructed Cu^2+^ g‐C3N4 and performed GSH depletion PDT. Among them, Cu^2+^ and GSH undergo oxidation‐reduction reactions, neutralizing GSH in tumor tissues and, thus, reducing the consumption of ROS by GSH. Furthermore, the product of the redox reaction can also catalyze the production of more ROS, which further enhances the killing effect of ROS on tumors during the treatment process.

At present, the commonly used GSH removal strategies are mainly Cu,^89^ Mn,^92^ Fe,^93^ or Au^94^ meta‐elements. However, excessive use of these metals may produce serious side effects such as systemic toxicity, which affects the conversion of GSH removal strategies to clinical.^95^, ^96^ The discovery of new substances that can consume GSH is an important issue that needs to be solved now. Liu^97^ and others designed an H_2_O_2_‐activated oxidative stress amplifier (OSA) to enhance the ROS generation of PDT by depleting GSH in tumor tissue. Cinnamaldehyde (Cin) and Ce6 are used as GSH scavengers and PSs, respectively, and they are assembled with ROS‐reactive amphiphilic polymer (DPL) to form DPL@CC micelles as OSA. Among them, Cin reacts specifically with thiol on GSH, which is a key functional group capable of scavenging ROS, resulting in the failure of GSH and applying it to GSH depletion. In the blood circulation, OSA can effectively protect Cin from albumin binding, thereby maintaining its GSH consumption capacity. Once OSA reaches the tumor site, high levels of H_2_O_2_ trigger the degradation of DPL and lead to the release of Cin and Ce6, which leads to GSH clearance and ROS levels up‐regulation. Compared with untreated cells, OSA down‐regulates GSH levels by about 18.9% and attenuates antioxidant ability of tumor cells. The experimental results show that this strategy of inhibiting the consumption of ROS by GSH achieves a 94% tumor suppression effect, confirming that Cin's strategy of inhibiting the ROS defense system can significantly enhance the killing of ROS to tumors. It can be seen that the strategy of suppressing the ROS defense system by reducing the GSH content helps to improve the treatment efficiency of ROS for tumors.

Furthermore, the researchers found that MTH1 in tumor cells prevents ROS from DNA damage to form a ROS defense system to protect tumor cells.^98^ Recently, an anti‐tumor method using MTH1 as an anti‐cancer target has been developed. Once the MTH1 inhibitor is combined with the active site of MTH1, it can successfully prevent the ROS defense system from weakening the ROS killing efficiency of the tumor and promoting tumor cell apoptosis.^99^ Zhao et al.^100^ used MTH1 inhibitors to hinder the ROS defense system to improve the lethality of ROS against tumors and achieved a good tumor suppression effect when ROS production was limited. The research team used SiO_2_ crosslinked nanomicelles as a carrier to deliver the MTH1 inhibitor TH588 together with the PS Ce6 to tumor tissue for PDT. It was found that TH588 and PDT have a synergistic effect, and the DNA damage inside the mitochondria and the nucleus is significantly increased, which significantly promotes the apoptosis of tumor cells. MTH1 inhibitors significantly improve the anti‐tumor effect of ROS. Therefore, the experiment confirmed that inhibiting the ROS defense system can enhance the killing ability of ROS on tumors, thereby enhancing the efficacy of PDT.

## 4 SUMMARY AND OUTLOOK

Photodynamic therapy has great potential in the field of tumor treatment due to its advantages such as high treatment efficiency and strong selectivity, and brings new opportunities for future tumor treatment. However, the obstacle of the TME to the efficacy of PDT cannot be ignored. ROS is the core to achieve the effect of inhibiting tumors. The harsh TME often does not allow a large amount of ROS in tumors. Therefore, PDT has not achieved the desired therapeutic efficiency in the process of tumor treatment. In the past few decades, researchers have been committed to improving the killing of tumors by ROS through the TME. This article reviews the latest developments in related research, including improving ROS production by alleviating tumor tissue hypoxia, developing oxygen‐independent efficient PDT, and inhibiting the ROS defense system. It is worth noting that, in addition to the strategy of improving the anti‐tumor ability of ROS in the TME, the study of increasing the penetration depth of excitation light and optimizing the process of photoinduced electron transfer and energy transfer has also improved ROS from the perspective of light source and PS. The above two aspects have been elaborated in relevant excellent reviews.^27^, ^101^, ^102^ The future development of PDT is inseparable from the application of nanotechnology. The combination of the nanodrug delivery system and PDT not only effectively solves the problems of PS hydrophobicity and systemic toxicity, and enhances the ability of PDT to target tumor tissues, but also provides the possibility for the realization of an integrated PDT with multi‐functionality, multi‐strategy combination, and multi‐therapeutic collaboration in the future.^103^, ^104^, ^105^, ^106^ For example, Liu et al.^107^ developed an H_2_O_2_/O_2_ self‐supplied nano‐agent (MSNs@CaO_2_‐ICG)@LA, which reacts with CaO_2_ and water to generate O_2_ and H_2_O_2_ to relieve tumor tissue hypoxia, and also uses MSNs to induce GSH depletion to protect ROS is protected from removal. However, although PDT has good application prospects, it has not yet become a first‐line treatment for tumors. In order to optimize PDT and promote its clinical translation and application, the following points should be paid attention to: (1) How to construct a PDT drug system with strong biocompatibility and photostability; (2) How to monitor the anti‐tumor efficiency of ROS to prevent excessive ROS killing effect from damaging normal tissues, and to achieve a balance between ROS killing efficiency and PDT safety; (3) During the construction of nanodrugs, it is necessary to systematically evaluate the toxicity, biodistribution, pharmacokinetics, targeting and intratumoral accumulation of nanocarriers; (4) Long‐term and repeated use of NIR light or X‐ray irradiation will inevitably damage normal tissues. Therefore, it is necessary to explore repeatable PDT with "clean" excitation sources^108^ to reduce related side effects.

## CONFLICT OF INTEREST

All authors declare that they have no conflicts of interest relevant to the submitted manuscript.

## AUTHOR CONTRIBUTION

Daozhen Chen and Rui Yang conceived the central idea; Lan Ming screened the literature and wrote the initial draft of the study; the remaining authors contributed to refining the ideas, carrying out additional analyses, and finalizing this study; all authors discussed and revised the manuscript.

## Data Availability

The data supporting this review come from previously published studies and datasets, which have been cited.
